# Empoderamiento y apoyo social en pacientes con enfermedad renal crónica: estudio de caso en Michoacán, México

**DOI:** 10.26633/RPSP.2017.164

**Published:** 2017-12-12

**Authors:** María Fernanda Carrillo Vega, Blanca Estela Pelcastre Villafuerte, Guillermo Salinas Escudero, Luis Durán Arenas, y Malaquías López Cervantes

**Affiliations:** 1 Departamento de Investigación de Epidemiología Clínica Instituto Nacional de Geriatría Ciudad de México México Departamento de Investigación de Epidemiología Clínica, Instituto Nacional de Geriatría, Ciudad de México, México.; 2 Centro de Investigación en Sistemas de Salud Centro de Investigación en Sistemas de Salud Cuernavaca México Centro de Investigación en Sistemas de Salud, Instituto Nacional de Salud Pública, Cuernavaca, México.; 3 Centro de Estudios Económicos y Sociales en Salud Hospital Infantil de México Federico Gómez Ciudad de México México Centro de Estudios Económicos y Sociales en Salud, Hospital Infantil de México Federico Gómez, Ciudad de México, México.; 4 Centro Nacional para la Salud de la Infancia y la Adolescencia Centro Nacional para la Salud de la Infancia y la Adolescencia Ciudad de México México Centro Nacional para la Salud de la Infancia y la Adolescencia, Ciudad de México, México.; 5 Dirección General de Planeación y Desarrollo en Salud Dirección General de Planeación y Desarrollo en Salud Ciudad de México México Dirección General de Planeación y Desarrollo en Salud, Ciudad de México, México.

**Keywords:** Poder, apoyo social, insuficiencia renal crónica, Power, social support, renal insufficiency, chronic, Poder, apoio social, insuficiência renal crónica

## Abstract

**Objetivo:**

*Identificar el nivel de empoderamiento y las características del apoyo social de los individuos con enfermedad renal crónica (ERC)*.

**Métodos:**

*Estudio transversal en personas con enfermedad renal crónica que habitan en el municipio de Hidalgo, Michoacán, México, y que asisten a la Asociación de Enfermos del Riñón, Asociación Civil (A.C.). Se indagó sobre el perfil sociodemográfico y las características personales asociadas a la enfermedad, el nivel de empoderamiento, el nivel de apoyo social y el funcionamiento familiar*.

**Resultados:**

*Cerca de 90% de la muestra cuenta con apoyo social suficiente, mientras que 40% de los participantes pertenece a familias semirrelacionadas o relacionadas. El puntaje de empoderamiento global fue de 117,5 ± 14,3; el empoderamiento comunitario fue más alto en el grupo de mayor edad (P < 0,05). La interacción social positiva es el componente del apoyo social que correlaciona con mayor fuerza con el nivel de empoderamiento (r = 0,333; P < 0,01)*.

**Conclusiones:**

*El empoderamiento está determinado por, y es un determinante del apoyo social y ha facilitado el acceso a la terapia de reemplazo renal en esta comunidad*.

## Abstract (en)

**Objective:**

Identify the degree of empowerment and the characteristics of the social support for individuals with chronic kidney disease (CKD).

**Methods:**

Cross-sectional study of people with chronic kidney disease living in the municipality of Hidalgo, Michoacán, Mexico, who belong to the Association of Kidney Patients, Civil Association. The study examined the sociodemographic profile and personal characteristics associated with the disease, the degree of empowerment, the degree of social support, and family functioning.

**Results:**

Close to 90% of the sample receives sufficient social support, while 40% of the participants belong to semi-cohesive or cohesive families The overall empowerment score was 117.5 ± 14.3; community empowerment was higher in the older group (*P* < 0.05). Positive social interaction is the social support component most strongly correlated with the degree of empowerment (*r* = 0.333; *P* < 0.01).

**Conclusions:**

Empowerment is determined by, and is a determinant of, social support and has facilitated access to renal replacement therapy in this community.

## RESUMO

**Objetivo:**

Identificar o nível de empoderamento e apoio social de pacientes com nefropatia crônica.

**Métodos:**

Estudo transversal realizado com pacientes com nefropatia crônica residentes no município de Fidalgo, Michoacán, México, que participavam de uma associação civil de pacientes renais crônicos (Asociación de Enfermos del Riñón). Foram investigados aspectos pessoais e sociodemográficos dos pacientes associados à doença, nível de empoderamento e apoio social e funcionamento familiar.

**Resultados:**

Cerca de 90% da amostra estudada dispõem de uma rede de apoio social satisfatória e 40% pertencem a famílias com boa coesão ou coesão média. A pontuação global de empoderamento foi de 117,5 ± 14,3 e o empoderamento comunitário foi maior no grupo com idade mais avançada (*P* < 0,05). Interação social positiva é o componente da rede de apoio social com correlação mais forte com o nível de empoderamento (*r* = 0,333; *P* < 0,01).

**Conclusões:**

O empoderamento é determinado pela rede de apoio social, sendo também um determinante deste apoio, e facilita o acesso à terapia renal substitutiva nesta comunidade.

La enfermedad renal crónica (ERC) es un problema de salud pública a nivel mundial con una prevalencia estimada de 13% (1). En México, las cifras exactas se desconocen debido a la carencia de un sistema nacional de registro; sin embargo, se han reportado prevalencias que oscilan entre 7% y 33% (2). Estas cifras han motivado el interés en el estudio de sus determinantes y su impacto, con el reporte de que, en muchas ocasiones, el retardo en su detección y tratamiento es una constante que afecta a los grupos más vulnerables. Al respecto, se ha reportado que la prevalencia de la ERC en poblaciones de muy bajos recursos en el territorio mexicano es de dos a tres veces mayor en comparación con la población general, y que se desconoce la causa de la enfermedad en aproximadamente 30% de los casos (3). Esto es reflejo de un sistema de salud carente de capacidad para responder a las necesidades de la población, en el cual el acceso a la atención se encuentra determinado por la afiliación a las instituciones de seguridad social, situación definida a su vez por la pertenencia al mercado laboral formal, de manera que los menos favorecidos tienen acceso limitado o nulo a los recursos para la atención de la enfermedad (4). Una tasa de mortalidad tres veces mayor en la población no afiliada es el dato que mejor refleja la gravedad del problema (5).

Por otra parte, quienes están afiliados suelen recibir atención de poca calidad. En ambos casos, los individuos se ven obligados a realizar acciones de diferente naturaleza para acceder a la atención de la enfermedad y así asegurar su supervivencia. Para que estas acciones sean exitosas, los individuos deben contar con ciertas características que los lleven a tener éxito en la tarea.

En este sentido, se ha documentado que una de las herramientas de mayor utilidad en esta búsqueda es el empoderamiento (6), que puede ser impulsado por o generador de apoyo social (7).

El empoderamiento es el proceso de toma de decisiones autónomas por medio del cual el individuo obtiene acceso a los recursos que lo ayudan a gestionar su condición actual y llevar a cabo acciones valiosas y significativas que impactan en su persona y contexto (8). En el ámbito de la salud, el empoderamiento permite al individuo obtener el control sobre su salud y mantener la integración social (9). Para que esto suceda, es necesaria la interacción del individuo con otros sujetos (10), específicamente con la red de relaciones sociales que lo rodea y que es percibida por este como significativa (11). Mediante esta red, el individuo obtendrá apoyo social, mecanismo que sirve como un “factor protector” contra la vulnerabilidad de los individuos frente a los efectos del estrés sobre la salud (12).

Pocos estudios han intentado clarificar la relación existente entre el empoderamiento y el apoyo social. En algunos de ellos (13) se presenta al apoyo social como una de las variables impulsoras del empoderamiento, al ser un recurso que debe controlarse para conseguir los objetivos y metas establecidas. Autores como Rosenfield, en cambio, apuntan hacia el empoderamiento como promotor del apoyo social, pues un individuo empoderado es más capaz de buscar apoyo en diferentes redes en comparación con quienes carecen de la capacidad para tomar el control de sus vidas (14).

El presente estudio tiene como objetivo identificar el nivel de empoderamiento y las características del apoyo social de los individuos con ERC que habitan en una comunidad en la que las acciones para obtener el tratamiento para la ERC se realizan de manera constante y en la que la red de apoyo parece jugar un papel determinante en este empoderamiento.

## MATERIALES Y MÉTODOS

### Diseño y población de estudio

Para alcanzar el objetivo del presente trabajo se llevó a cabo un estudio transversal descriptivo en la Asociación de Enfermos del Riñón A.*C*. de Ciudad Hidalgo, Municipio de Hidalgo, Michoacán, México.

Este municipio se caracteriza por tener la mayor prevalencia de ERC en el estado. Es esta localidad se han registrado aproximadamente 1 000 casos, lo que representa 20% del total a nivel estatal. En Ciudad Hidalgo, cabecera del municipio, se halla la Asociación de Enfermos del Riñón A.C., cuya actividad se centra en brindar apoyo emocional e instrumental a personas con la enfermedad en sus diferentes etapas, sobre todo a aquellas personas que ya cuentan con terapia de reemplazo renal (TRR). De acuerdo con su censo, cerca del 10% del total de personas con ERC en el Municipio de Hidalgo (aproximadamente 134 individuos), residen en la cabecera del municipio.

La elección de esta área responde a la necesidad de explorar el tema desde un entorno caracterizado por la presencia de desigualdades en salud, cuya alta prevalencia de ERC y la carencia de recursos para la atención a la enfermedad obligan a las personas que sufren esta enfermedad a tomar el control de los recursos con los que cuentan para poder acceder al tratamiento de la enfermedad. Esta característica parece responder a un proceso de empoderamiento, que ha trascendido en la creación de una de las pocas asociaciones civiles en México destinadas a esta población**.

El tamaño de muestra fue calculado mediante la fórmula para la estimación de una proporción en una población, tomando en cuenta que en el censo de la Asociación se tiene registrado un total de 134 personas con ERC y esperando que por lo menos un 23% de las personas tuvieran un nivel de empoderamiento medio. Considerando los datos reportados por Pick y cols. (15), el tamaño de muestra fue de 69 personas, conseguido mediante muestreo aleatorio simple. Se incluyeron a las personas con diagnóstico confirmado de ERC entre 18 y 85 años de edad, con o sin TRR, que decidieran participar de manera voluntaria y firmaran la carta de consentimiento informado.

### Recolección de datos

Las autoridades de la asociación realizaron contacto directo con los miembros, seleccionados de manera aleatoria, para convocarlos a una reunión informativa, cuya finalidad fue dar a conocer las características del estudio, los objetivos, la metodología, y el tiempo que duraría la aplicación de los cuestionarios. Al finalizar la presentación de la investigadora principal, se realizó una sesión de dudas, tras lo cual se realizaron citas para aplicar los instrumentos.

### Identificación del perfil sociodemográfico

La información se recolectó mediante un cuestionario elaborado ex profeso para el presente estudio. Se incluyeron preguntas acerca de las características individuales y familiares, la afiliación a instituciones o programas de salud, el nivel socioeconómico, los antecedentes patológicos familiares y personales de importancia relacionados con la ERC. Se incluyeron preguntas sobre el tiempo con el diagnóstico con la ERC, el personal de salud que lo diagnosticó y le proporciona tratamiento en la actualidad, el tipo de TRR, el tiempo que lleva con TRR; se incluyeron preguntas para indagar acerca de la recepción de información y capacitación sobre la enfermedad y sus implicaciones.

### Nivel de empoderamiento

El nivel de empoderamiento se determinó mediante la Escala de Agencia Personal y Empoderamiento (ESAGE). Consta de 42 reactivos agrupados en las siguientes variables: autoeficacia, autodeterminación, control sobre mis conductas, pensamiento independiente, identificación de necesidad de cambio, miedo al éxito, reconocimiento de mi aprendizaje, percepción de mi contexto y control sobre mi entorno. Las cuatro opciones de respuesta (“nunca”, “casi nunca”, “casi siempre”, “siempre”) se presentan en escala tipo Likert. Dicha escala fue elaborada y validada en población mexicana (15) y se obtuvieron valores de alfa de Cronbach cercanas a uno (agencia: α = 0,718; empoderamiento: α = 0,749).

Antes de su aplicación, se explicaron los objetivos y características del instrumento, de tal manera que la escala pudiera ser autoaplicada. En aquellos casos en los que el participante no supiera leer o escribir, la escala fue completada con apoyo del encuestador.

### Apoyo social

El estudio de la red de apoyo social se realizó mediante dos escalas de autoaplicación que, al igual que con la ESAGE, fueron explicadas con detenimiento a los participantes y completadas por el encuestador en caso de que el participante así lo solicitara. Se aplicó la encuesta de apoyo social retomada del Estudio de resultados médicos (MOS, por sus siglas en inglés), para pacientes con enfermedades crónicas (16). Se trata de un cuestionario breve y autoadministrado, que consta de 20 reactivos, cada uno con respuestas en escala Likert de cinco opciones (casi siempre, muchas veces, a veces sí y a veces no, pocas veces, casi nunca) mediante las cuales se evalúa el apoyo social en cuatro dimensiones: emocional e informacional, instrumental, interacción social positiva y apoyo afectivo. Los autores del instrumento reportaron valores de alfa de Chronbach cercanos a uno: apoyo emocional e informacional (α = 0,96), apoyo instrumental (α = 0,92), interacción positiva (α = 0,94), apoyo afectivo (α = 0,91) (16). El instrumento se ha validado en varios países de América Latina y se obtuvieron valores muy similares (17, 18).

Además, se utilizó la Escala de Evaluación de la Cohesión y Adaptabilidad Familiar (FACES III) 3° versión (19, 20), que ha sido validada en población mexicana obteniendo valores de alfa de Chronbach cercanos a 1 (α = 0,70). La escala original consta de 40 reactivos, cada uno con una escala Likert de cinco opciones (casi siempre, muchas veces, a veces sí y a veces no, pocas veces, casi nunca), divididos en dos partes. Para fines del presente trabajo solo se aplicó la sección que evalúa el nivel de cohesión (familia no relacionada, semirrelacionada, relacionada y aglutinada) y flexibilidad de la familia (rígida, estructurada, flexible y caótica) tal como el sujeto la percibe en ese momento.

A cada participante se le proporcionó la descripción detallada del estudio, tanto en forma verbal como escrita; en esta última se incluyó la descripción del estudio, los objetivos, beneficios y posibles riesgos. Se les solicitó su firma, con la que manifestaron su consentimiento informado. Todos los datos obtenidos se mantendrán como confidenciales.

El presente estudio se considera de mínimo riesgo, debido a que no se utilizaron procedimientos invasivos. Para su realización se siguieron los preceptos de la declaración Helsinki y Reglamento de la Ley General de la Salud en materia de investigación para la salud.

El estudio contó con la aprobación del comité de investigación y ética de la Universidad Nacional Autónoma de México, registro 143/2014.

### Análisis estadístico

La información recabada fue analizada mediante el paquete estadístico STATA v.13.1. Se obtuvieron la media y la desviación estándar para las variables continuas, y frecuencias y porcentajes para las variables categóricas. Se realizó comparación de medias mediante t de Student y comparación de proporciones por medio de la x2 de Pearson. Se determinó la correlación de las variables mediante correlación de Pearson.

## RESULTADOS

Participaron en estudio un total de 69 pacientes, de los cuales 57% (*n* = 39) era hombre (cuadro 1). La mediana de edad fue de 36 años (intervalo de confianza de 95% 26,9–47,1); cerca de 71% de los menores de 36 años era soltero; la misma proporción de casados se observó en los mayores de 36 años. Cerca de 40% de la población se hallaba en el nivel socioeconómico D, caracterizado porque los inmuebles pueden ser propios, pero con carencia de los principales bienes y servicios satisfactores. Del 32% (*n* = 22) de individuos que cuenta con seguridad social, el 43% (*n* = 15) es menor de 38 años.

La media de tiempo con la ERC fue de 5,3 ± 5,7 años y con terapia de reemplazo renal de 2,9 ± 3,3 años. La principal TRR fue la hemodiálisis, sobre todo en los mayores de 38 años, mientras que el trasplante fue más frecuente en los más jóvenes. Cerca de 60% de la muestra, en su mayoría los participantes de menor edad, hace uso de servicios como nutrición y psicología; principalmente dentro de las instalaciones de los servicios de salud, pues la asociación no cuenta con este tipo de servicios.

Se observó que cerca de 90% de la muestra cuenta con apoyo social suficiente en todas las esferas, con un puntaje medio de 71,4 ± 12,8 (cuadro 2). De los participantes, 36% pertenece a familias semirrelacionadas o relacionadas y cerca de 60% pertenece a familias caóticas; no se observaron diferencias significativas por grupo de edad en ninguna de las escalas. El puntaje de empoderamiento global (cuadro 3) fue de 117,5 ± 14,3; no se observaron diferencias por grupo de edad en torno al empoderamiento individual; sin embargo, el empoderamiento comunitario fue más alto en el grupo de mayor edad (*P* < 0,05). Se observó que el locus de control es la dimensión del empoderamiento que muestra diferencias por grupo de edad (*P* < 0,05).

Como se observa en el cuadro 4, la interacción social positiva es el componente del apoyo social que correlaciona con mayor fuerza con el nivel de empoderamiento (*r* = 0,333; *P* < 0,01), seguido por el apoyo emocional (*r* = 0,245, *P* < 0,05).

## DISCUSIÓN

El presente estudio representa uno de los primeros acercamientos al fenómeno del empoderamiento y la red social en pacientes con ERC en el contexto mexicano. Los resultados demuestran que el empoderamiento individual es un proceso presente en los participantes, que trasciende al nivel comunitario principalmente entre los individuos de mayor edad, lo que con base en la teoría de McMillan (21, 22) nos lleva a pensar en la presencia de un mayor sentimiento de pertenencia y arraigo en este segmento de la muestra. La red social principal es la familia que, de acuerdo con los participantes, se caracteriza por ser una red carente de roles y de liderazgo. A pesar de esta peculiaridad, el apoyo social recibido es percibido por los individuos como adecuado.

El nivel adecuado de empoderamiento guarda una relación estrecha con la pertenencia a la asociación. En este sentido, el acercamiento a este tipo de grupos de apoyo denota la capacidad del individuo para plantear y lograr objetivos, dimensiones que forman parte del empoderamiento individual. Por otro lado, los individuos que acceden a estos grupos son provistos con herramientas que incrementan capacidades como el autocontrol, la toma de decisiones informada y otras propias del empoderamiento.

**CUADRO 1.** Características generales de la población por grupo de edad^a^

	Total		≤36 años		>36 años		P
	*n* = 69	%	*n* = 35	%	*n* = 34	%	
Sexo						
Femenino	30	43,5	15	42,9	15	44,1	0,916
Masculino	39	56,5	20	57,1	19	55,9	
Religión						
Católica	67	97,1	35	1,0	32	94,1	0,145
Otra religión	2	2,9	0	0	2	5,9	
Estado civil						
Soltero	29	42,7	24	70,6	5	14,7	0,000
Casado	33	48,5	9	26,5	24	70,6	
Viudo	3	4,4	0	0	3	8,8	
Unión libre	3	4,4	1	2,9	2	5,9	
Nivel socioeconómico						
E	3	4,4	1	2,9	2	5,9	0,022
D	26	37,7	7	20	19	55,9	
D+	18	26,1	11	31,4	7	20,6	
C-	12	17,4	10	28,6	2	5,9	
C	9	13,0	5	14,3	4	11,8	
C+	1	1,5	1	2,9	0	0,0	
Antecedentes familiares de importancia						
HTA	5	7,3	3	8,6	2	5,9	0,962
DM	16	23,2	7	20	9	26,5	
HTA+DM	15	21,7	8	22,9	7	21,0	
HTA+ERC	2	2,9	1	2,9	1	2,9	
DM+ERC	10	14,5	8	22,9	2	5,9	
HTA+DM+ERC	6	8,7	3	8,6	3	8,8	
Sin antecedentes	15	21,7	5	14,3	10	29,4	
Diagnósticos relacionados con la ERC						
HTA+DM	40	58,0	19	54,3	21	61,8	0,536
Sin diagnósticos relacionados	29	42,0	16	45,7	13	38,2	
Tiempo con el diagnóstico (años)	5,3 ± 5,7		5,5 ± 5,1		5,1 ± 6,2		0,74
Lugar donde recibe atención para la ERC						
Hospital de secretaría de salud	41	59,4	18	51,4	23	67,7	0,212
Hospital o clínica de seguridad social	22	31,9	15	42,9	7	20,6	
Hospital o clínica privada	4	5,8	1	2,9	3	8,8	
Asociación de enfermos del riñón	2	2,9	1	2,9	1	2,9	
Terapia de reemplazo renal (%)	38	55,2	12	34,3	26	76,5	
Hemodiálisis	13	18,8	11	31,5	2	5,9	0,002
Trasplante	9	13,0	6	17,1	3	8,8	
Diálisis peritoneal	9	13,0	6	17,1	3	8,8	
Tiempo con terapia de reemplazo renal (años)	2,9 ± 3,3		3,7 ± 3,5		2,3 ± 2,9		0,107
Médico que lo atiende en la actualidad						
Nefrólogo	43	62,3	20	57,1	23	67,7	0,659
Médico familiar o general	17	24,6	10	28,6	7	20,6	
Otro	9	13,0	5	14,3	4	11,8	
Recibe atención o asesoría de otros profesionales						
Nutriólogo	20	29,0	7	20,0	13	38,2	0,279
Nutriólogo + psicólogo	26	37,7	14	40,0	12	35,3	
Médico clínico	2	2,9	1	2,9	1	2,9	
Psicólogo + médico clínico	2	2,9	0	0,0	2	5,9	
Ninguno	19	27,5	13	37,1	6	17,7	
Información sobre la ERC (%)						
Se le brinda	62	89,9	29	82,9	33	97,1	0,052
No se le brinda	7	10,1	6	17,1	1	2,9	
Lugar donde se brinda información (%)						
Hospital de secretaría de salud	36	58,1	15	51,7	21	63,6	0,687
Hospital o clínica de seguridad social	14	22,6	8	27,6	6	18,2	
Hospital o clínica privada	3	4,8	1	3,5	2	6,1	
Asociación de enfermos del riñón	9	14,5	5	17,2	4	12,1	
Esta información le ha ayudado a mejorar (%)						
Siempre	47	75,8	23	79,3	24	72,7	0,443	
Casi siempre	9	14,5	5	17,2	4	12,1	
Algunas veces	5	8,1	1	3,5	4	12,1	
Casi nunca	1	1,6	0	0,0	1	3,0	

a Los valores de *P* para la comparación por grupos de edad se presentan como promedio ± desviación estándar; los datos categóricos se presentan como frecuencia (porcentaje). HTA, hipertensión arterial: DM, diabetes mellitus; ERC, enfermedad renal crónica.

Nivel socioeconómico: E, carece de todos los servicios y bienes satisfactores; D, cuenta con una propiedad pero carece de diversos servicios y satisfactores; D+, tiene cubierta la mínima infraestructura sanitaria de su hogar; C-, tiene cubiertas las necesidades de espacio y sanidad y cuenta con los enseres y equipos que le aseguran el mínimo de practicidad y comodidad en el hogar; C, alcanza un nivel de vida práctica y con ciertas comodidades, y cuenta con una infraestructura básica en entretenimiento y tecnología; C+, tiene cubiertas todas las necesidades de calidad de vida, sin embargo tiene ciertas limitantes para invertir y ahorrar para el futuro.

Este último constructo ha sido ampliamente estudiado en contextos caracterizados por la presencia constante de desigualdades en la distribución de recursos (23, 24). A pesar de que México se caracteriza por ser un país de marcada inequidad (25–27), poco se ha explorado acerca del proceso de empoderamiento y de cómo es posible alcanzarlo a partir de los recursos que la red le proporciona al individuo con el objetivo de acceder al tratamiento de las enfermedades.

De acuerdo con nuestros resultados, la TRR más frecuente entre los individuos de la asociación fue la hemodiálisis. Este dato es difícil de comparar con otros a nivel internacional debido a las diferencias entre los sistemas de salud, y a nivel nacional debido a la carencia de un registro, por tipo de lugar de atención y por comunidad, sea rural o urbana. No obstante, nuestros resultados difieren de los reportados por Méndez- Durán y cols. (28) para el Instituto Mexicano del Seguro Social (IMSS) y por la Sociedad Latinoamericana de Nefrología e Hipertensión (29), quienes reportan que la TRR de mayor uso a nivel nacional es la diálisis peritoneal (DP). Podemos atribuir esta diferencia a las características de los servicios de salud que atienden a esta población pues, como se observa, cerca de 60% de individuos reciben atención en hospitales de la Secretaría de Salud, dependencias que no cuentan con las facilidades para proporcionar DP y que subrogan el servicio de hemodiálisis; por este motivo, los individuos pagan una cuota proporcional a su capacidad de pago.

Por otra parte, se observa que los hospitales de seguridad social atienden a cerca de 32% de las personas con ERC. Esta cifra está por debajo de lo reportado por García-García y cols. (5), quienes encontraron que en Jalisco, México, 72% de las personas en TRR reciben atención en hospitales de seguridad social. Esta diferencia está determinada en gran medida por la carencia de aseguramiento en salud, consecuencia del tipo de actividad económica principal en la región, que es la agricultura seguida por el comercio informal. Esto implica que los individuos tengan que ser atendidos por los hospitales de la secretaría de salud y pagar una cuota proporcional a su ingreso mensual (30). Por último, se puede observar que el mayor porcentaje de individuos atendidos en instituciones de seguridad social son los menores de 36 años, lo que lleva a pensar que son ellos quienes acceden con mayor facilidad al campo laboral formal. Cabe destacar que esta comunidad se caracteriza por una alta prevalencia de ERC en personas jóvenes, lo cual difiere con los datos nacionales, donde la media de edad de presentación de la enfermedad se acerca a los 50 años (2). Estos hallazgos sugieren la necesidad de estudiar las características fisiopatológicas de la enfermedad en esta región y los factores de riesgo, tanto individuales, como ambientales, socioculturales y económicos, que pueden estar involucrados en su alta prevalencia en la población joven.

En el presente estudio, 60% de las personas atendidas en hospitales de la Secretaría de Salud cuenta con los servicios de consulta y hemodiálisis; sin embargo, no se cubren los medicamentos. Este rubro corre a cargo de los individuos y sus familias, lo que en la mayoría de los casos lleva al empobrecimiento. Entre otras, esta es una de las razones por las cuales es probable encontrar poca adherencia, lo que aumenta el riesgo de complicaciones y muerte. El panorama no suele ser mejor para quienes cuentan con servicios de salud, pues la carencia de infraestructura y de personal de salud capacitado limitan la entrega de medicamentos y servicios y encarecen la calidad de la atención (31).

Cualquier sean la condición laboral y del aseguramiento en salud, el enfrentamiento de la enfermedad a nivel físico y psicológico supone un reto para los individuos pues, en la mayoría de los casos, se ven obligados a buscar estrategias para acceder al tratamiento y mejorar sus condiciones de salud. La movilización de recursos individuales, grupales y comunitarios es esencial en este proceso; destaca como recurso inherente al individuo la red de apoyo social, en especial la familiar.

Según los participantes, el apoyo social está presente en forma adecuada, el apoyo emocional es el tipo más frecuente y se correlaciona de manera importante con el empoderamiento. Se observó una correlación positiva muy significativa entre la interacción social positiva y el empoderamiento que, con base a lo establecido por Hall y cols. (11) y por Rosenfield (14), nos hace pensar que el apoyo social positivo podría estar relacionado con la capacidad de los individuos para empoderarse y acceder al tratamiento y ser, a su vez, un determinante del bienestar del individuo como lo reportaron Göz y cols. (32) y Wen y cols. (33). Esta relación deberá ser estudiada con mayor profundidad en el futuro.

Nuestros resultados nos orientan a calificar al apoyo social brindado por la familia como un apoyo negativo que, a pesar de ser fuente de conflicto en un número elevado de ocasiones, puede impulsar a buscar otras fuentes de apoyo social. Este hallazgo está estrechamente ligado con la correlación encontrada entre interacción social positiva y empoderamiento y concuerda con una de las teorías de empoderamiento psicológico más importantes que es la de Zimmerman (10, 34), quien apunta que uno de los componentes fundamentales del empoderamiento es el interactivo.

**CUADRO 2.** Apoyo social y funcionamiento familiar en pacientes con ERC^a^

	Media ± DE		Media ± DE		Media ± DE		P
	Total (*n* = 69)		≤36 años (*n* = 35)		>36 años (*n* = 34)		
Puntaje en la escala de apoyo social	71,4 ± 12,8		72,5 ± 13,6		70,2 ± 12,0		0,443
Apoyo emocional	31,8 ± 7,6		33,0 ± 7,4		30,5 ± 7,6		0,18
Apoyo instrumental	17,6 ± 3,6		17,6 ± 3,9		17,6 ± 3,2		0,969
Apoyo afectivo	13,3 ± 2,9		13,1 ± 3,3		13,5 ± 2,4		0,589
Interacción social positiva	17,5 ± 3,4		17,8 ± 3,5		17,2 ± 3,4		0,448
Puntaje en la escala de funcionamiento familiar						
Adaptabilidad	40,9 ± 6,3		41,1 ± 6,5		40,7 ± 6,1		0,785
Cohesión	30,7 ± 7,6		28,5 ± 7,1		32,9 ± 7,6		0,015
	*n* = 69	%	*n* = 35	%	*n* = 34	%
Apoyo global							0,305
Apoyo escaso	9	13,0	6	17,1	3	8,8	
Apoyo suficiente	60	87,0	29	82,9	31	91,2	
Apoyo emocional							0,326
Apoyo escaso	13	18,8	5	14,3	8	23,5	
Apoyo suficiente	56	81,2	30	85,7	26	76,5	
Apoyo instrumental							0,97
Apoyo escaso	6	8,7	3	8,6	3	8,8	
Apoyo suficiente	63	91,3	32	91,4	31	91,2	
Apoyo afectivo							0,414
Apoyo escaso	6	8,7	4	11,4	42	5,6	
Apoyo suficiente	63	91,3	31	88,6	32	94,1	
Interacción social positiva							0,983
Apoyo escaso	2	2,9	1	2,9	1	2,9	
Apoyo suficiente	67	97,1	34	97,1	33	97,1	
Cohesión familiar							0,388
Familia no relacionada	6	8,7	3	8,6	3	8,8	
Familia semirrelacionada	25	36,2	10	28,6	15	44,1	
Familia relacionada	25	36,2	16	45,7	9	26,5	
Familia aglutinada	13	18,8	6	17,1	7	20,6	
Adaptabilidad familiar							0,177
Familia rígida	6	8,7	5	14,3	1	2,9	
Familia estructurada	13	18,8	7	20,0	6	17,7	
Familia flexible	9	13,0	6	17,1	3	8,8	
Familia caótica	41	59,4	17	48,6	24	70,6	

a Los valores de *P* para la comparación por grupos de edad se presentan como promedio ± desviación estándar; los datos categóricos se presentan como frecuencia (porcentaje).

**CUADRO 3.** Características del empoderamiento por grupo de edad^a^

	Total	≤36 años	>36 años	P
	n = 69	n = 35	n = 34	
Índice de empoderamiento	117,5 ± 14,3	116,0 ± 16,9	119,1 ± 11,9	0,385
Empoderamiento individual	99,9 ± 11,2	99,9 ± 2,3	99,8 ± 8,1	0,982
Empoderamiento comunitario	17,7 ± 5,6	16,1 ± 5,2	19,3 ± 5,6	0,019
Dimensiones del empoderamiento individual			
Poder para ejercer control	28,2 ± 3,5	27,8 ± 3,6	28,8 ± 3,3	0,199
Poder para la elección	35,8 ± 5,4	34,9 ± 4,5	36,6 ± 6,1	0,176
Poder para el cambio	19,7 ± 3,1	19,3 ± 3,2	20,0 ± 3,0	0,268
Locus de control	17,9 ± 3,8	16,9 ± 3,3	19,0 ± 4,1	0,026

a Los valores de *P* para la comparación por grupos de edad se presentan como promedio ± desviación estándar.

**CUADRO 4.** Correlaciones entre empoderamiento total y categorías de apoyo social y funcionamiento familiar

Variable	Coeficiente	P
Apoyo social global	0,2593	0,0314
Apoyo emocional	0,2448	0,0426
Apoyo instrumental	-0,0011	0,9926
Apoyo afectivo	0,1986	0,1019
Interacción social positiva	0,3326	0,0052
Adaptabilidad	0,2308	0,0564
Cohesión	0,1931	0,112

Dadas las características de la población y en acuerdo con nuestros resultados, concluimos que el apoyo social es una herramienta clave para el empoderamiento. La evidencia científica que precede a este trabajo nos permite plantear que la presencia de ambos constructos en niveles adecuados constituye un elemento clave para enfrentar las vicisitudes de la ERC. Por otra parte, sugerimos que tanto el apoyo social como el empoderamiento forman parte de la maquinaria indispensable para controlar los recursos disponibles, acceder a los sistemas de salud y participar de manera activa y junto con el personal de salud en la toma de decisiones, sobre todo en contextos caracterizados por inequidades pronunciadas.

Una de las limitaciones más importantes de nuestro estudio es que la muestra elegida no permite hacer generalizaciones, pues la extracción de la muestra de una población que por su naturaleza posee características propias del empoderamiento, la hace diferente al resto de pacientes con ERC. Por otra parte, mediante el acercamiento elegido no es posible identificar elementos como las fuentes y mecanismos de obtención de la información, los procesos de análisis de la esta con respecto a la realidad individual y social, la toma de decisiones conscientes, si el empoderamiento ha transformado estructuras de poder resultando en la mejora de las oportunidades de atención a la ERC a nivel individual y en toda la población, y los mecanismos de participación de la red de apoyo social en estos procesos. Además, el diseño dificulta el estudio de la direccionalidad de la asociación existente entre el apoyo social y el empoderamiento.

Con el objetivo de conocer más a fondo el fenómeno del empoderamiento, los mecanismos de acción de la red de apoyo y su impacto social, se sugiere que en futuras investigaciones se aborde el problema desde una perspectiva fenomenológica, mediante metodologías cualitativas que permitan indagar las vivencias y experiencias de los individuos.

### Declaración

Las opiniones expresadas en este manuscrito son responsabilidad del autor y no reflejan necesariamente los criterios ni la política de la RPSP/PA- JPH y/o de la OPS.
